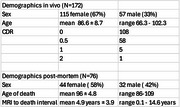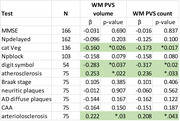# MR‐visible perivascular space burden associated with concurrent cognition and post‐mortem assessment of cerebrovascular disease

**DOI:** 10.1002/alz70856_107686

**Published:** 2026-01-09

**Authors:** David Lahna, Daniel Schwartz, Natalie E Roese, Justin Hurworth, Randy L Woltjer, Nora Mattek, Jeffrey A Kaye, Lisa C Silbert

**Affiliations:** ^1^ NIA‐Layton Oregon Alzheimer's Disease Research Center, Oregon Health & Science University, Portland, OR, USA; ^2^ NIA‐Layton Aging & Alzheimer's Disease Center, Portland, OR, USA; ^3^ Advanced Imaging Research Center, Oregon Health & Science University, Portland, OR, USA; ^4^ NIA‐Layton Aging & Alzheimer's Disease Research Center; Neuropathology Core, Oregon Health & Science University, Portland, OR, USA

## Abstract

**Background:**

MRI‐visible white matter (WM) perivascular space (PVS) burden is associated with aging as well as with various disease states, including Alzheimer's Disease (AD) and vascular cognitive impairment (VCI). The objective of this study was to examine relationships between PVS in subcortical WM with tissue pathologies associated with AD and VCI and premortem neuropsychological test performance.

**Method:**

172 subjects received a 1.5T MRI including coronal SPGR and spin echo PD/T2 as part of research at the Oregon Alzheimer's Disease Research Center (OADRC). All participants underwent neuropsychological testing, and a subset (*n* = 76) had NACC pathology data available (Table 1).

MRI volumes were resampled to 1mm isotropic resolution. The T1 was skull stripped, denoised, and registered to a synthetic FLAIR volume generated by taking the product of the PD and T2. Tissue types were segmented (Freesurfer 7.1.1) from the skull stripped and denoised T1 volume. PVS segmentation was accomplished using a local heterogeneity approach on NAWM (non‐synFLAIR hyperintense) voxels in T_1_‐weighted volumes with object‐level morphology constraints used to identify likely MRI‐visible PVS.

Multiple linear regressions were performed using PVS burden metrics as independent variables with cognitive tests as dependent variables while controlling for age, sex and brain volume (for PVS predictors) and the time interval between in vivo MRI and death (for pathology predictors)

**Result:**

Mean age at MRI and death were 86.6 and 96.0 years, respectively. Greater WM PVS burden (count and volume) was related to poorer performance on tests of frontal executive function (Trails B, Category Fluency, digit symbol) and increased pathological markers of both large and small vessel cerebrovascular disease (Table 2). Total PVS was not related to memory (delayed recall), global cognition (MMSE) or AD pathology.

**Conclusion:**

I*n vivo* MRI‐visible PVS burden within the subcortical WM is associated with a cognitive profile and neuropathologic findings consistent with cerebrovascular disease, supporting its use as a vascular marker in aging studies. Future work should consider longitudinal analysis to confirm the temporal order of increases in MR‐visible PVS burden and cognitive decline.